# Supplementary material for: From sequence to enzyme mechanism using multi-label machine learning
Source: BMC Bioinformatics. 2014 May 19;15:150. doi: 10.1186/1471-2105-15-150 (PMC4229970; doi:10.1186/1471-2105-15-150)
Supplement: Additional file 2 — Java code of ml2db. Additional file ml2db_code.tar.gz contains the Java source code to run the multi-label machine learning experiments and save the results to database. The code’s Javadoc is included. [file 1471-2105-15-150-S2.zip › additional file 2/ml2db/ml2db/doc/main/ml2db/al/utility/class-use/UtilityMetric.html]

Uses of Class main.ml2db.al.utility.UtilityMetric


JavaScript is disabled on your browser.


- Overview
- Package
- Class
- Use
- Tree
- Deprecated
- Index
- Help

- Prev
- Next

- Frames
- No Frames

- All Classes

## Uses of Class main.ml2db.al.utility.UtilityMetric

- Packages that use UtilityMetric

  | Package | Description |
  | --- | --- |
  |  |  |
  | --- | --- |
  | main.ml2db.al.learning |  |
  | main.ml2db.al.utility.confidence |  |
  | main.ml2db.al.utility.globaldistance |  |
  | main.ml2db.al.utility.hybrid |  |
  | main.ml2db.al.utility.neighbours |  |
  | main.ml2db.al.utility.pickers |  |
  | main.ml2db.al.utility.pickers.byfrequency |  |
  | test.ml2db.al |  |
- - ### Uses of UtilityMetric in main.ml2db.al.learning

    Methods in main.ml2db.al.learning that return UtilityMetric

    | Modifier and Type | Method and Description |
    | --- | --- |
    |  |  |
    | --- | --- |
    | `UtilityMetric` | ExperimentDetails.`getUtilityMetric()` |

    Constructors in main.ml2db.al.learning with parameters of type UtilityMetric

    Constructor and Description

    `ExperimentDetails(IdMultiLabelInstances unlabelled,
    MulanLearner algorithm,
    java.lang.String distanceFunction,
    DbManagers dbManagers,
    UtilityMetric utilityMetric,
    java.lang.String imagesPath)`

    With set of unlabelled instances (no arff record identifier)

    `ExperimentDetails(int labelledArffId,
    int unlabelledArffId,
    MulanLearner algorithm,
    java.lang.String distanceFunction,
    DbManagers dbManagers,
    UtilityMetric utilityMetric,
    java.lang.String imagesPath)`

    Both labelled and unlabelled arff record identifier.

    `ExperimentDetails(int unlabelledArffId,
    MulanLearner algorithm,
    java.lang.String distanceFunction,
    DbManagers dbManagers,
    UtilityMetric metric,
    java.lang.String imagesPath)`

    Only Unlabelled arff record identifier.
  - ### Uses of UtilityMetric in main.ml2db.al.utility.confidence

    Subclasses of UtilityMetric in main.ml2db.al.utility.confidence

    | Modifier and Type | Class and Description |
    | --- | --- |
    |  |  |
    | --- | --- |
    | `class` | `ConfidenceMetric` Combines a Positioning metric (a metric that considers positively if an instance is close to other unlabelled instances and distant from other labelled instances) with the average certainty of the instance prediction (that is, how distant the average of confidence over all labels is from 50%) |
  - ### Uses of UtilityMetric in main.ml2db.al.utility.globaldistance

    Subclasses of UtilityMetric in main.ml2db.al.utility.globaldistance

    | Modifier and Type | Class and Description |
    | --- | --- |
    |  |  |
    | --- | --- |
    | `class` | `GlobalDistanceMetric` Positioning metric: a metric that considers positively if an instance is close to other unlabelled instances and distant from other labelled instances. |
  - ### Uses of UtilityMetric in main.ml2db.al.utility.hybrid

    Subclasses of UtilityMetric in main.ml2db.al.utility.hybrid

    | Modifier and Type | Class and Description |
    | --- | --- |
    |  |  |
    | --- | --- |
    | `class` | `GlobalDistanceWithConfidenceMetric` Combines a Positioning metric (a metric that considers positively if an instance is close to other unlabelled instances and distant from other labelled instances) with the average certainty of the instance prediction (that is, how distant the average of confidence over all labels is from 50%) |
  - ### Uses of UtilityMetric in main.ml2db.al.utility.neighbours

    Subclasses of UtilityMetric in main.ml2db.al.utility.neighbours

    | Modifier and Type | Class and Description |
    | --- | --- |
    |  |  |
    | --- | --- |
    | `class` | `NeighbourhoodNetwork` A collection of instances "needed" to label other instances. |
  - ### Uses of UtilityMetric in main.ml2db.al.utility.pickers

    Subclasses of UtilityMetric in main.ml2db.al.utility.pickers

    | Modifier and Type | Class and Description |
    | --- | --- |
    |  |  |
    | --- | --- |
    | `class` | `LabelRandomPicker` Class |
    | `class` | `RandomAttributePicker` Picks an attribute uniformly randomly and then one of the unlabelled instances having that attribute. |
    | `class` | `RandomAttributeSetPicker` Class |
    | `class` | `RandomInstancePicker` Class |
    | `class` | `RandomLabelsPicker` Class |
    | `class` | `RandomNeigbourhoodPicker` Class |
    | `class` | `RandomPicker` Class |
  - ### Uses of UtilityMetric in main.ml2db.al.utility.pickers.byfrequency

    Subclasses of UtilityMetric in main.ml2db.al.utility.pickers.byfrequency

    | Modifier and Type | Class and Description |
    | --- | --- |
    |  |  |
    | --- | --- |
    | `class` | `AttributesByFrequencyPicker` Class |
    | `class` | `AttSetByFrequencyPicker` Serves the instances in order of frequency of their attribute sets (most frequent first). |
    | `class` | `ByFrequencyPicker` Picks each of the features (class, attribute or attribute set) by descending order of frequency, and then, uniformly randomly, one of the instances of that feature. |
    | `class` | `LabelsByFreqPicker` Class |
    | `class` | `NearestNeighboursByFreqUtility` Class |
  - ### Uses of UtilityMetric in test.ml2db.al

    Methods in test.ml2db.al with parameters of type UtilityMetric

    | Modifier and Type | Method and Description |
    | --- | --- |
    |  |  |
    | --- | --- |
    | `static ExperimentDetails` | ExperimentDetailsTest.`generateExperimentManager(int unlabelledArffId, UtilityMetric metric)` |

- Overview
- Package
- Class
- Use
- Tree
- Deprecated
- Index
- Help

- Prev
- Next

- Frames
- No Frames

- All Classes
